# Iron–Integrated Nitrogen–Rich Nanocarriers Boost Symbiotic Nitrogen Fixation and Growth in Soybean (*Glycine max*)

**DOI:** 10.3390/nano15181453

**Published:** 2025-09-21

**Authors:** Taiming Zhang, Weichen Zhao, Muhammed Nadeem, Usama Zaheer, Yukui Rui

**Affiliations:** 1 College of Resources and Environmental Sciences, China Agricultural University, Beijing 100193, China; 2 Professor Workstation in Wuqiang, China Agricultural University, Wuqiang 053300, China; 3 State Key Laboratory for Environmental Chemistry and Ecotoxicology, Research Center for Eco–Environmental Sciences, Chinese Academy of Sciences, Beijing 100085, China

**Keywords:** Fe_2_O_3_/g–C_3_N_4_ nanocomposite, symbiotic nitrogen fixation, nanofertilizer, iron nutrition, soybean, sustainable agriculture

## Abstract

Global food security is challenged by population growth and the environmental toll of conventional fertilizers. Enhancing biological nitrogen fixation (BNF) in legumes like soybean (*Glycine max*) is a sustainable fertilization alternative. This study investigates a graphitic carbon nitride/iron oxide (Fe_2_O_3_/g–C_3_N_4_ or FC) nanocomposite as a dual–functional fertilizer to improve iron (Fe) nutrition and BNF in soybeans. A pot experiment was conducted using different FC concentrations (10, 100, and 200 mg kg^−1^), alongside controls. Results showed that the 100 mg kg^−1^ FC treatment (FC2) was most effective, significantly increasing soybean biomass, nodule number, and nodule fresh weight. The FC2 treatment also enhanced photosynthetic rates and chlorophyll content (SPAD values) while reducing stomatal conductance and transpiration, indicating improved water–use efficiency. Furthermore, FC application bolstered the plant’s antioxidant system by increasing the activity of superoxide dismutase (SOD) and peroxidase (POD). Elemental analysis confirmed that FC treatments significantly increased the uptake and translocation of Fe and nitrogen (N) in plant tissues. These findings demonstrate that the FC nanocomposite acts as a highly effective nanofertilizer, simultaneously addressing iron deficiency and boosting nitrogen fixation to promote soybean growth. This work highlights its potential as a sustainable solution to enhance crop productivity and nutrient use efficiency in modern agriculture.

## 1. Introduction

Despite record global cereal production, food security remains threatened by conflicts, extreme weather, and resource inefficiencies. Enhancing crop productivity sustainably is thus critical. Soybean, a crucial crop for global food security, derives significant nutritional value from its high protein (35–45%) and oil (17–25%) content [1,2]. Its cultivation relies heavily on nitrogen (N) fertilization, but conventional N fertilizers exhibit low efficiency (30–60%), with significant losses via volatilization, leaching, and runoff. These losses contribute to environmental degradation, including eutrophication, soil acidification, and greenhouse gas emissions [3,4,5,6,7,8]. Improving nitrogen use efficiency (NUE) is therefore essential for sustainable agriculture [9,10].

Biological nitrogen fixation (BNF) through the symbiotic relationship between legumes and rhizobia offers a sustainable alternative [11,12,13,14]. One important natural resource is the abundance of atmospheric nitrogen (78% N_2_). In soybean, symbiotic nitrogen fixation (SNF) can supply 50–80% of the plant’s nitrogen needs by converting atmospheric N_2_ into ammonia within root nodules [15,16,17]. This anaerobic process relies on a hypoxic microenvironment safeguarded by leghemoglobin, which protects the oxygen–sensitive nitrogenase enzyme [18]. Enhancing SNF efficiency is thus paramount for reducing synthetic fertilizer dependence and advancing agroecological sustainability.

Iron (Fe) is a critical micronutrient in this symbiosis. Beyond its roles in chlorophyll biosynthesis, photosynthesis, and electron transport [19,20,21,22], iron is a key component of leghemoglobin in nodules. It facilitates oxygen transport to bacterial respiratory chains while preventing nitrogenase inactivation [23,24]. Recent research reveals iron’s regulatory role in nodulation itself. An iron–sufficient state stabilizes GmBTS, which positively regulates the transcription factor NSP1, ultimately enhancing the expression of key nodulation genes like GmNIN. This signaling cascade increases nodule density and nitrogenase activity [25], positioning iron availability as a direct governor of symbiotic efficiency.

Nanotechnology is emerging as a transformative force in agriculture, enhancing NUE, addressing agro–environmental challenges, and increasing productivity [26,27]. Nano–fertilizers and nano–pesticides offer sustainable alternatives to conventional practices. Their nanoscale dimensions enable superior plant uptake, resulting in enhanced efficacy at lower application rates compared to chemical counterparts [28]. This improves yield and nutrient utilization while reducing environmental contamination [29]. Nanotechnology presents innovative strategies to augment nutrient use efficiency, and both iron–based and carbon–based nanomaterials show promise. Nano–Fe_2_O_3_ applications (e.g., foliar sprays at 30–50 mg/L) have been shown to enhance soybean photosynthesis, biomass, and yield [30,31]. Meanwhile, the metal–free polymer graphitic carbon nitride (g–C_3_N_4_) is prized for its low cost, ease of synthesis, and environmental friendliness [32,33]. While primarily explored for water purification, its terrestrial applications are emerging [34]. g–C_3_N_4_ can mitigate heavy metal stress in plants and is known to stimulate growth by enhancing enzyme activity and improving soil nutrient cycling [35,36,37]. It represents a sustainable nitrogen–enriched carbon nanostructure.

While both pure nano–Fe_2_O_3_ and g–C_3_N_4_ offer individual benefits, their composite—Fe_2_O_3_/ g–C_3_N_4_ (FC)—remains underexplored in agricultural contexts. Nano–Fe_2_O_3_ alone improves nodulation and yield but may lack synergistic effects with carbon structures. g–C_3_N_4_ enhances enzyme activity and nutrient uptake but does not provide essential iron nutrients [38]. The FC composite potentially combines these advantages: Fe_2_O_3_ serves as an iron nutrient source and nanozyme, while g–C_3_N_4_ improves electron transfer and stress resilience [25,39,40,41]. Moreover, the photocatalytic nitrogen fixation mechanism in FC mirrors biological nitrogen fixation in soybeans—both processes are energy–intensive and iron–dependent. In FC, Fe_2_O_3_ provides active sites for nitrogen adsorption, analogous to iron’s role in nitrogenase cofactors (FeMo–co) and leghemoglobin [42,43].

This study investigates FC as a dual–functional nanofertilizer designed to simultaneously enhance iron supply and biological nitrogen fixation in soybeans. We hypothesize that FC supplementation will improve nodulation, nitrogenase activity, and yield by leveraging synergistic effects between Fe_2_O_3_ and g–C_3_N_4_. By comparing FC with pure nano–Fe_2_O_3_ and g–C_3_N_4_, we aim to demonstrate its superiority in boosting SNF efficiency and NUE, ultimately contributing to sustainable soybean production.

## 2. Materials and Methods

### 2.1. Characterization of Fe_2_O_3_/g–C_3_N_4_

The FC used in this study was synthesized via the electrostatic self–assembly method [44]. The g–C_3_N_4_ (50–700 nm, thickness 0.6–3 nm, purity greater than 99.98%, the forbidden band width is 2.7 eV [45], CAS number 143334-20-7, Zhongye New Materials Co., Ltd. (Jinhua, China)) and nano–Fe_2_O_3_ (30 nm purity greater than 99.95%, CAS number 1309-37-1, Zhongke New Materials Co., Ltd. (Luohe, China)) employed for synthesis. A homogeneous dispersion was prepared by ultrasonically treating 900 mg g–C_3_N_4_ in deionized water (40 mL, 40 kHz, 200 W) for 30 min, during which the pH was adjusted to 6.0 using 0.2 mM H_2_SO_4_. Subsequently, 100 mg Fe_2_O_3_ nanoparticles was added, and the mixture underwent magnetic stirring (600 rpm) for 4 h to ensure uniform integration. The resulting composite was isolated via rotary evaporation, followed by sequential washing with methanol and deionized water (3× each) to remove residual ions and organics. Finally, the product was dried at 80 °C for 24 h to yield the g–C_3_N_4_/Fe_2_O_3_ composite.

**Figure 1:**
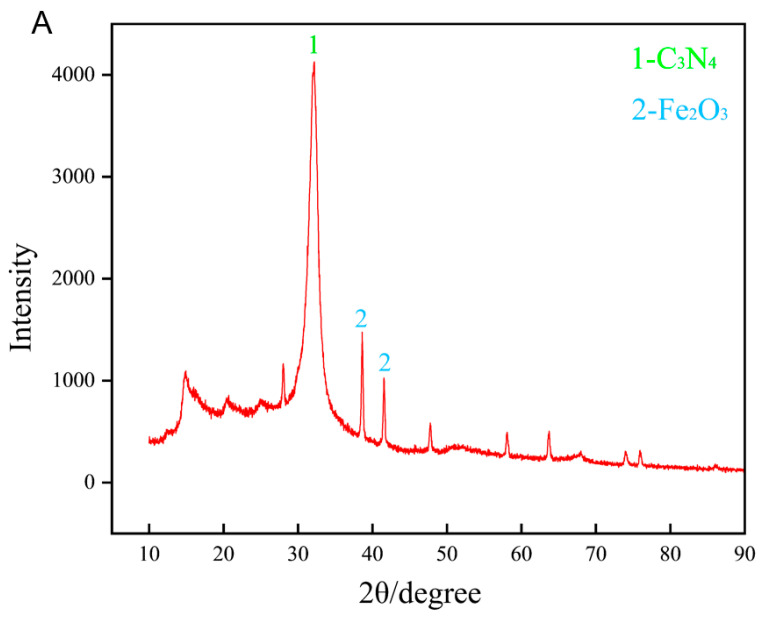

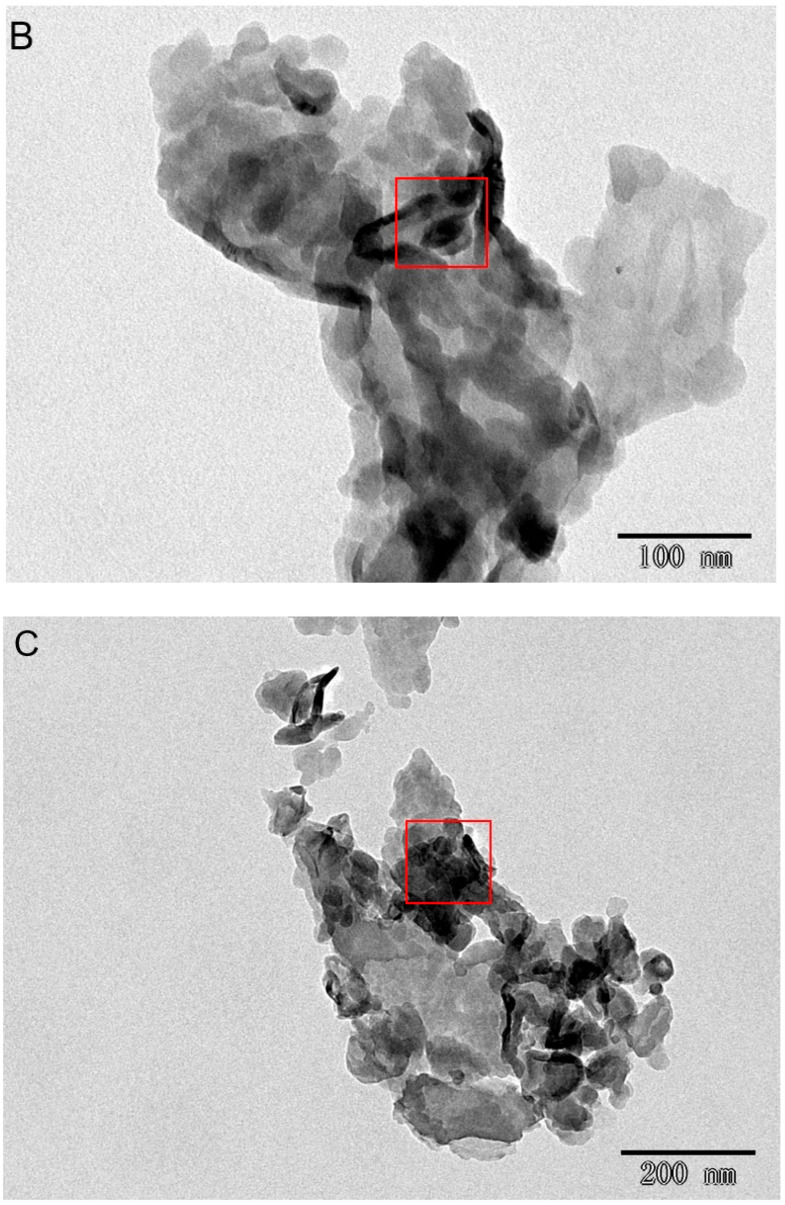

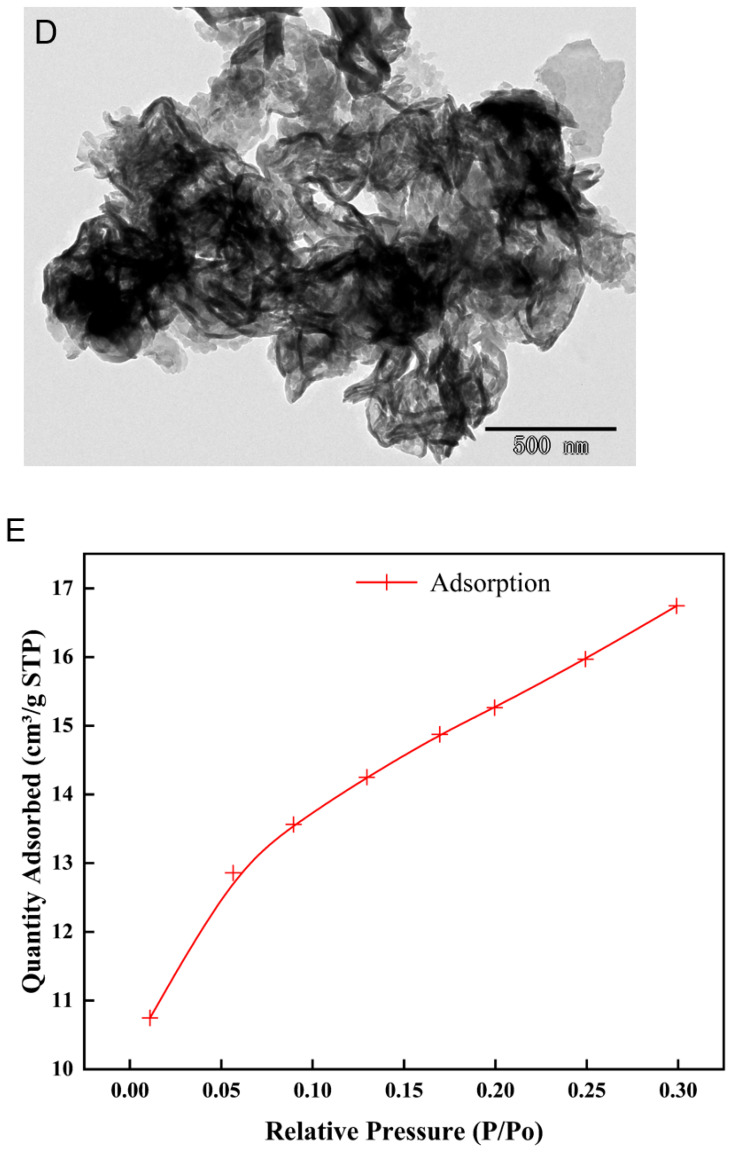

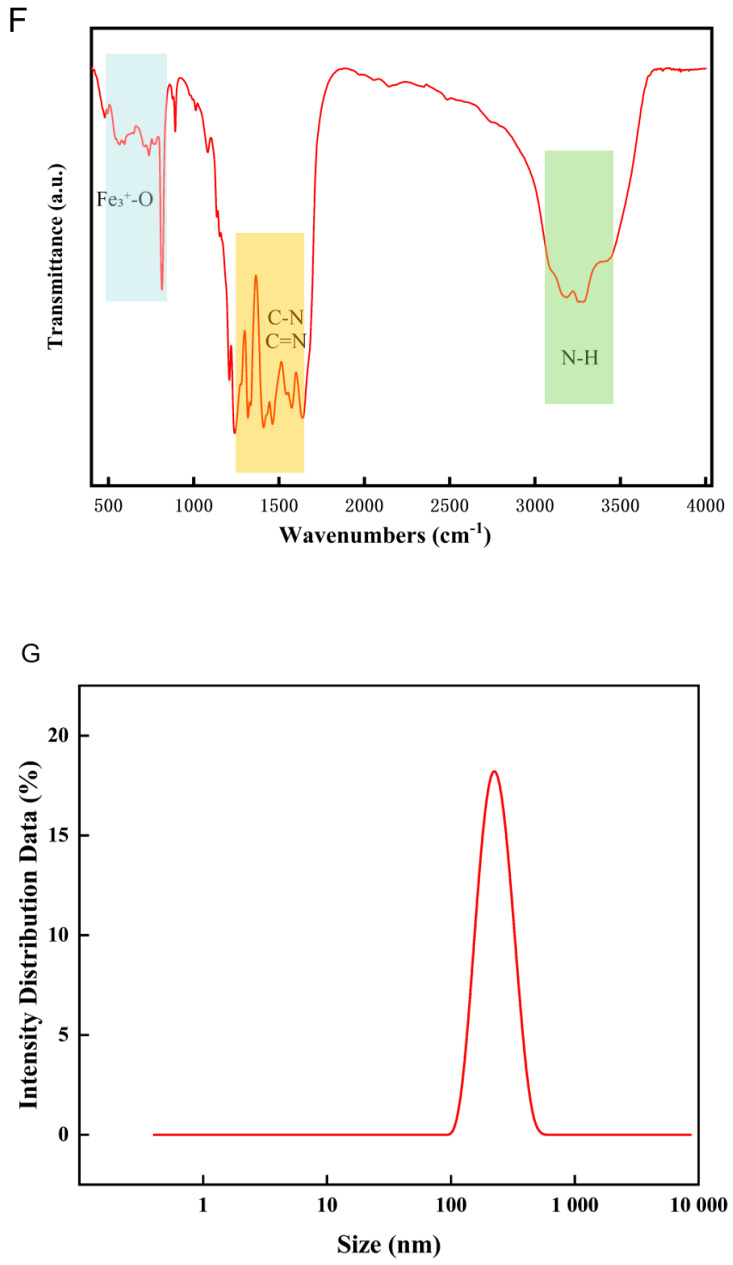

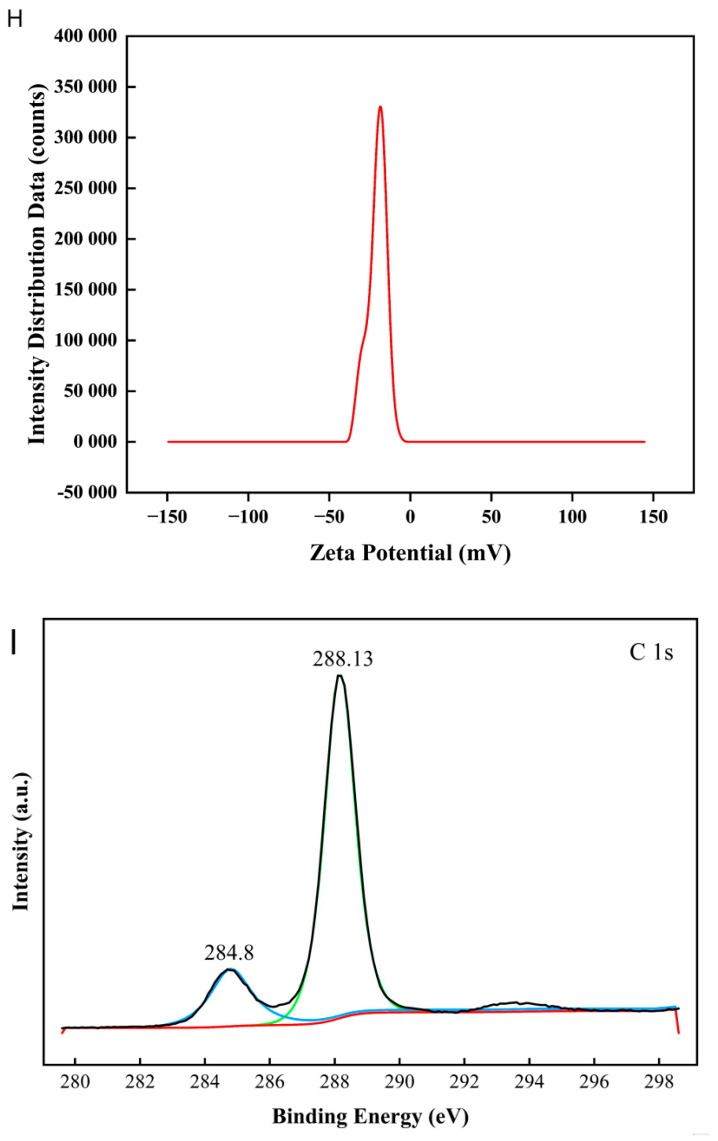

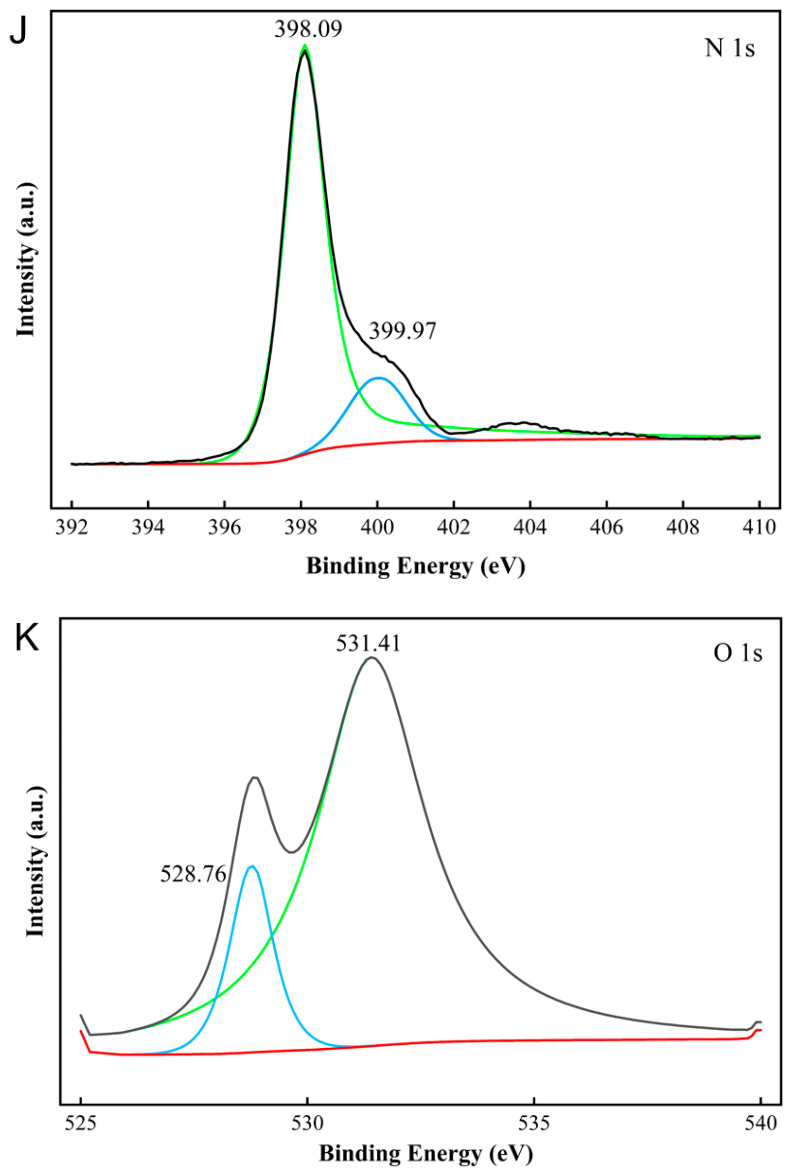

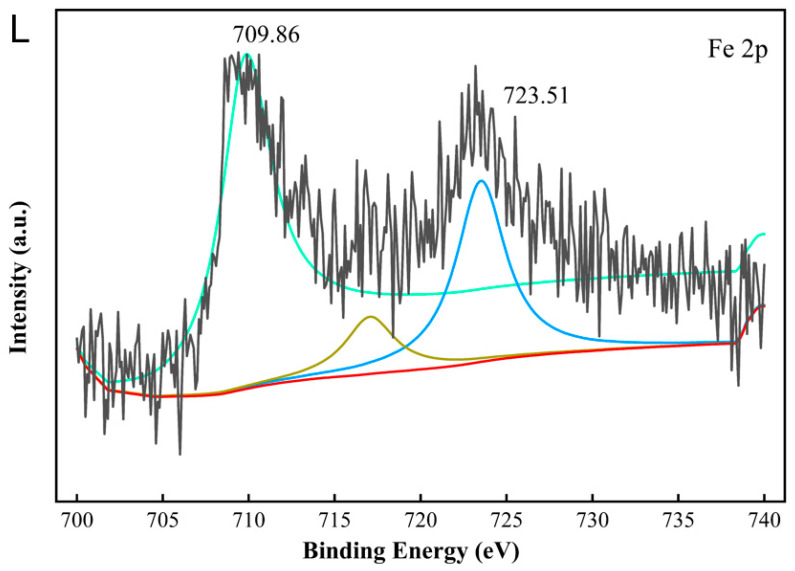
The X–ray diffraction (XRD) pattern of FC (**A**). Transmission electron microscopy (TEM) images of FC, with scale bars of 100 nm (**B**), 200 nm (**C**), and 500 nm (**D**), respectively, the red box indicates that Fe_2_O_3_ is stacked on top of g–C_3_N_4_. Nitrogen adsorption isotherm (**E**). The FTIR spectrum (**F**). Size Distribution (**G**). Zeta Distribution (**H**). High–resolution XPS spectra of the (**I**) C 1s, (**J**) N 1s, (**K**) O 1s, and (**L**) Fe 2p regions of samples.

A comprehensive physicochemical characterization of FC was conducted to elucidate its structural features, which are essential for assessing its potential in agricultural applications (Figure 1). The X–ray diffraction (XRD) pattern of the g–C_3_N_4_/Fe_2_O_3_ composite. The dominant peak marked “1” at around 27° corresponds to the (002) plane of g–C_3_N_4_, while the smaller peaks marked “2” at approximately 33° and 36° are indicative of Fe_2_O_3_ crystal planes, confirming the presence of both components within the composite (Figure 1A). Figure 1B–D present transmission electron microscopy (TEM) images at varying magnifications. In Figure 1B (scale bar: 100 nm), dark Fe_2_O_3_ nanoparticles are uniformly anchored onto the sheet-like g–C_3_N_4_ matrix, reflecting strong interfacial integration. Figure 1C (scale bar: 200 nm) highlights localized nanoparticle aggregation on the layered surface, whereas Figure 1D (scale bar: 500 nm) shows the overall architecture composed of folded nanosheets forming an interconnected three–dimensional network. The hierarchical structure of the FC composite, with its increased surface area and porosity, is primarily advantageous for its function as a fertilizer. This structure likely enhances the dispersion and retention of nutrients in the rhizosphere, facilitates the slow release of iron ions, and improves the contact interface with plant roots or soil microorganisms, thereby potentially promoting nutrient uptake and utilization efficiency. The comprehensive physical adsorption analysis (Figure 1E), performed using a TriStar II 3020 instrument (Version 3.02) (Norcross, GA, USA) with nitrogen as the adsorbate at a constant bath temperature of −195.85 °C, yielded significant insights into the sample’s surface characteristics, revealing a BET surface area of 51.1202 ± 0.8426 m^2^/g as calculated from multipoint data within the relative pressure (P/Po) range of 0.056 to 0.249, which confirms a high specific surface area indicative of potential applications in catalysis or adsorbent materials. The adsorption isotherm demonstrated Type II behavior, characterized by a gradual increase in adsorbed quantity from 10.7473 cm^3^/g STP at P/Po = 0.011 to 16.7442 cm^3^/g STP at P/Po = 0.299, suggesting a non–porous or macroporous structure without significant hysteresis, as visually supported by the linear plot. The FTIR spectrum of the Fe_2_O_3_/g–C_3_N_4_ composite (Figure 1F) exhibits characteristic absorption bands confirming the presence of both components. A broad band centered at approximately 3281 cm^−1^ is attributed to the stretching vibrations of N–H groups in the g–C_3_N_4_ framework. The distinct peaks between 1200 and 1650 cm^−1^, notably at 1637 and 1573 cm^−1^, correspond to the typical stretching modes of the C–N and C=N heterocyclic rings in g–C_3_N_4_. Furthermore, the sharp peak at around 813 cm^−1^ is assigned to the out–of–plane bending vibration of tri–s–triazine units. The presence of Fe_2_O_3_ is indicated by the absorption band below 600 cm^−1^, which is associated with the Fe–O stretching vibration. The Fe_2_O_3_/g–C_3_N_4_ nanocomposite was characterized for particle size distribution and zeta potential to assess its colloidal properties (Figure 1G,H). Size analysis via dynamic light scattering (DLS) revealed a Z–average diameter of 222.5 nm with a low polydispersity index (PDI) of 0.048, indicating narrow size homogeneity; the cumulative distribution showed D10, D50, and D90 values of 159 nm, 230 nm, and 334 nm, respectively, reflecting a moderate size range and unimodal distribution, as visualized in the intensity–based plot. Additionally, zeta potential measurements in aqueous dispersion yielded a mean value of −20.8 mV and a standard deviation of 4.01 mV, demonstrating moderate negative surface charge and good colloidal stability suitable. The XPS analysis of the Fe_2_O_3_/g–C_3_N_4_ nanocomposite (Figure 1I–L) reveals its definitive chemical composition and interfacial interactions. The C 1s spectrum exhibits two characteristic peaks at 284.8 eV and 288.13 eV, attributable to sp^2^ C–C bonds and N–coordinated carbon (N–C=N) in the g–C_3_N_7_ phase, respectively. Concurrently, the N 1s spectrum is deconvoluted into primary components at 398.09 eV (C–N=C) and 399.97 eV (N–(C)_3_), further confirming the successful formation of g–C_3_N_4_. The O 1s spectrum presents a dominant lattice oxygen peak of Fe_2_O_3_ at 528.76 eV alongside a surface oxygen/hydroxyl species peak at 531.41 eV. Finally, the Fe 2p spectrum displays distinct peaks at 709.86 eV (Fe 2p_3_/_2_) and 723.51 eV (Fe 2p_1_/_2_) with accompanying satellite features, unequivocally identifying the presence of Fe^3+^ in the α–Fe_2_O_3_ phase. The slight but consistent chemical shifts observed in the core–level spectra, compared to the pristine components, indicate strong electronic coupling at the heterojunction interface, likely facilitated by Fe–O–C or Fe–N–C bonding, which is crucial for enhancing the material’s functional properties.

### 2.2. Experimental Design

The pot experiment was carried out in a greenhouse at the West Campus of China Agriculture University (CAU). Soybean seeds (cultivar “Zhonghuang 13,” sourced from Taobao) underwent surface sterilization with a 5% NaClO solution for 5 min. The seeds were then rinsed with deionized water to eliminate any remaining chemicals. Original soil was collected from the Shangzhuang Experimental Station of CAU (40.139659° N, 116.181222° E), air–dried under ambient conditions, and subsequently sieved through a 2 mm mesh, the soil physicochemical properties shown in Table 1. Based on Freitas et al. [46], macro– and micronutrients were incorporated at the following rates (mg kg^−1^ soil): P, 50 (Ca [H_2_PO_4_]_2_) and 60 (KH_2_PO_4_); K, 80 (KH_2_PO_4_); S, 40 (MgSO_4_·7H_2_O); B, 1.0 (H_3_BO_3_); Co, 0.1 (CoSO_4_·7H_2_O); and Mo, 0.5 ([NH_4_]_6_Mo_7_O_24_·4H_2_O). Eight treatments were established, each with 2 kg soil per pot (mg kg^−1^ soil): CK, control; CK2, CO(NH_2_)_2_ (60.20) + EDTA–Fe (45.32); Fe, nano–Fe_2_O_3_ (10); CN, C_3_N_4_ (90); F + C, nano–Fe_2_O_3_ (10) + C_3_N_4_ (90); FC1, FC2, and FC3, with FC applied at 10, 100, and 200, respectively. Soil was equilibrated for 14 days under greenhouse conditions to stabilize its physical and chemical properties, thereby creating a consistent environment for plant growth. Before sowing, four seeds were planted per pot to ensure adequate plant density for subsequent analysis. Notably, the seeds were not inoculated with rhizobia; instead, nodulation relied entirely on the native soil microbiota. This approach was intentionally designed to assess the inherent symbiotic capabilities of indigenous rhizobia populations and to better investigate the effects of nanomaterials on nodule development. Seedlings were thinned to one per pot 15 days after emergence, and samples were collected at 60 days.

### 2.3. Measurement of Soybean Phenotypes

After 60 days, plants were harvested and processed as follows: roots were rinsed sequentially with deionized water and HNO_3_ to remove adsorbed ions, followed by separation of roots, shoots, and root nodules. Morphometric parameters (root, shoot, nodule count) and fresh weights (roots, shoots, nodules) were recorded using an electronic balance. Soybean root and leaf samples were oven–dried to constant weight, with dry biomass subsequently quantified [47].

### 2.4. Chlorophyll Quantification and Gas Exchange Analysis

After 60 days of growth, gas exchange parameters were quantified to assess photosynthetic efficiency and stomatal regulation: net photosynthetic rate (Pn) (carbon assimilation capacity), stomatal conductance (Gs) (stomatal aperture regulation and CO_2_ diffusion), transpiration rate (Tr) (water vapor flux and cooling capacity), and vapor pressure deficit (Vpdl) (leaf–to–air humidity gradient driving transpiration). Measurements employed an LI–6400XT photosynthesis system (LI–Cor, Lincoln, NE, USA) on the third fully expanded sun–exposed leaf. Measurements were performed on undisturbed foliage during daylight hours, specifically targeting the third fully expanded apical leaf from the canopy apex. A chlorophyll meter (Konic Minolta, Tokyo, Japan) was used to record SPAD values at ten points close to the main vein after leaves from the same position were chosen for the chlorophyll content [48]. The average of these readings was used to represent the relative chlorophyll content.

### 2.5. Lipid Peroxidation Analysis and Antioxidant Enzyme Activities

Using commercial assay kits (Nanjing Jiancheng Bioengineering Institute, Nanjing, China), the homogenates were centrifuged at 8000× *g* for 10 min at 4 °C. The supernatants were then examined to determine the levels of malondialdehyde (MDA) and the activities of superoxide dismutase (SOD), peroxidase (POD), and catalase (CAT) [49]. SOD activity was measured using a xanthine/xanthine oxidase system following the kit protocol, with absorbance at 560 nm indicating the extent of nitroblue tetrazolium (NBT) reduction. POD activity was quantified by monitoring guaiacol oxidation to tetraguaiacol at 470 nm, with absorbance recorded at 0 and 10 min to calculate reaction rates [50]. CAT activity was assessed based on H_2_O_2_ decomposition, where residual peroxide reacted with ammonium molybdate to form a yellow complex; absorbance at 405 nm was inversely proportional to enzyme activity [51]. MDA levels were determined using the thiobarbituric acid (TBA) method, with absorbance measured at 532 nm and corrected at 600 nm for background interference. Concentrations were calculated using the molar extinction coefficient [52].

### 2.6. Elemental Composition Analysis

Following Zhou’s methodology [53], elemental composition of soybean leaves, stems, and roots was analyzed. A two–step dehydration procedure was applied to the dried specimens: first, they were heated to 105 °C for 30 min, and then they were dried at 75 °C while maintaining their weight. An elemental analyzer (Vario EL, Elementar, Langenselbold, Germany) was used to measure the amount of nitrogen (N) and carbon (C) in the finely powdered, dried material. For multi–element analysis, ~0.2 g aliquots were digested in high–purity concentrated HNO_3_ (8 mL) in vented tubes for 12 h under dark incubation. Samples underwent microwave–assisted digestion (MARS6, CEM, Buckingham, UK) using a three–stage temperature program: 120 °C (30 min), 140 °C (3 h), and 170 °C until around 1 mL of fluid was obtained. Digestates were diluted to 50 mL with ultrapure water, filtered through 0.25 μm PTFE membranes, and adjusted to appropriate dilutions. ICP–MS analysis (Elan DRC–e platform, PerkinElmer, San Diego, CA, USA) was employed to determine elemental concentrations with high analytical precision.

### 2.7. Statistical Analysis

Data are presented as means ± standard deviation (SD) derived from four biological replicates (n = 4). Inter–group comparisons were performed via one–way analysis of variance (ANOVA) with Tukey’s multiple comparisons test. Significant differences relative to control treatments are indicated: * *p* < 0.05, ** *p* < 0.01, *** *p* < 0.001, **** *p* < 0.0001.

## 3. Result and Discussion

### 3.1. Phytoeffects on Soybeans

**Figure 2:**
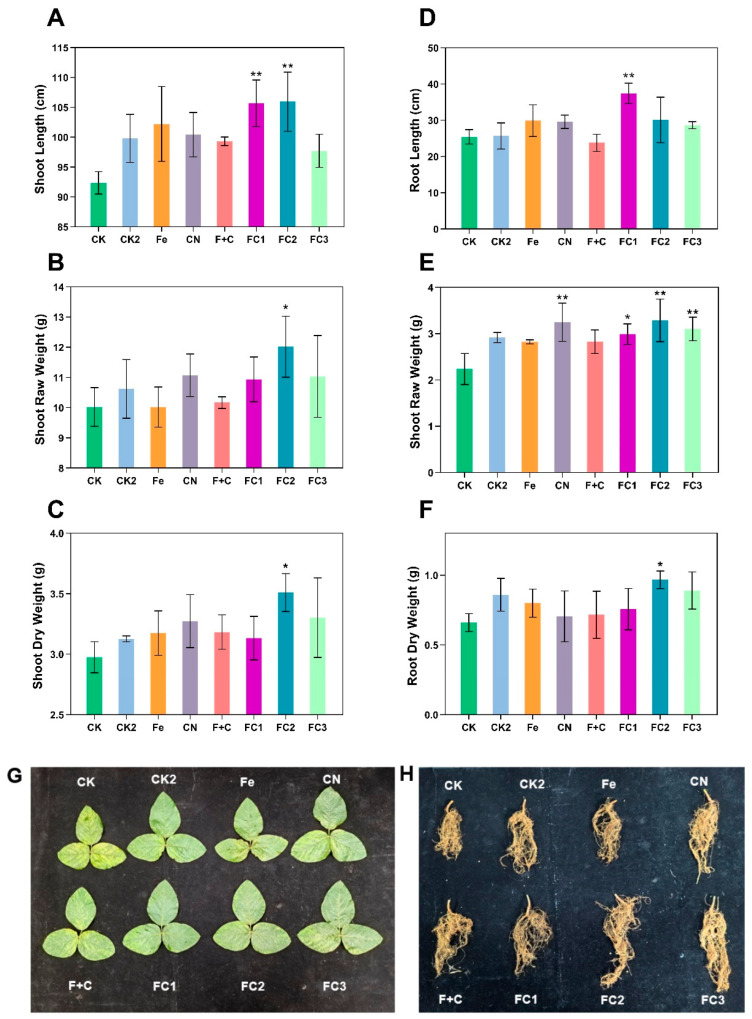
Effects on (**A**) Primary root elongation, (**B**) Root fresh biomass accumulation, (**C**) Root dry matter content, (**D**) Shoot vertical extension, (**E**) Shoot fresh biomass yield, (**F**) Shoot desiccation–resistant biomass. (**G**) Foliar structural characteristics, (**H**) Root system topology. The data points for each treatment group were collected from four independent biological replicates (n = 4). The error bars represent the standard error of the mean (SEM). Asterisks (*) indicate statistically significant differences compared to the control group: * *p* < 0.05, ** *p* < 0.01, *** *p* < 0.001, **** *p* < 0.0001.

The impact of FC on soybean growth characteristics was assessed, with key findings visually summarized in Figure 2. Regarding aerial biomass development, Figure 2A revealed that shoot length was significantly increased under FC1 and FC2 treatments, rather than FC3. Meanwhile, Figure 2B,C demonstrated that shoot fresh weight and dry weight were most markedly enhanced by FC2 and FC3, outperforming the control (CK) and other treatments. In terms of root architecture, Figure 2D indicated pronounced root elongation in the FC1 group, whereas FC3 showed a more moderate increase compared to CK. Additionally, Figure 2E confirmed elevated root fresh weight under FC1 and FC3, and Figure 2F highlighted a statistically significant increase in root dry weight with FC2 and FC3. Morphological validation in Figure 2G,H further supported these trends, showing improved leaf expansion and denser root systems in FC–treated plants compared to CK. Statistical analysis indicated significant effects for FC1, FC2, and FC3, underscoring FC’s potential role in promoting plant development, enhancing nitrogen fixation, and improving iron supply. These observations suggest that FC may function effectively as a dual–purpose nanofertilizer to support soybean growth, particularly at moderate to higher concentrations (e.g., 100–200 mg kg^−1^).

### 3.2. The Impact on Nodules

**Figure 3:**
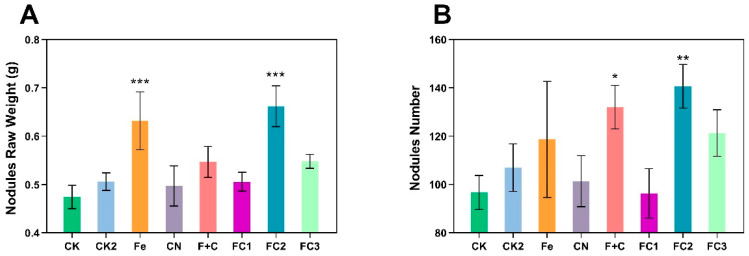
The impact of root nodule fresh weight (**A**) and root nodule quantity (**B**). Asterisks (*) indicate statistically significant differences compared to the control group: * *p* < 0.05, ** *p* < 0.01, *** *p* < 0.001, **** *p* < 0.0001.

The effects of FC on soybean nodulation are quantified in Figure 3. Figure 3A demonstrates that FC2 and FC3 treatments significantly increased nodule fresh weight compared to controls and other treatments (*p* < 0.05), with asterisks highlighting statistical superiority. Figure 3B further reveals a marked elevation in nodule number under FC2 treatment (*p* < 0.01), while FC3 also showed increased nodulation albeit with reduced significance. Although FC1 and F + C exhibited moderate improvements, their effects were less pronounced than FC2. Nodules from FC2–treated plants displayed denser proliferation and larger morphology relative to CK groups. Collectively, these data underscore FC2 as an optimal strategy for enhancing soybean nodulation efficiency.

### 3.3. Effects on Photosynthetic Pigments and Gas Exchange Parameters

**Figure 4:**
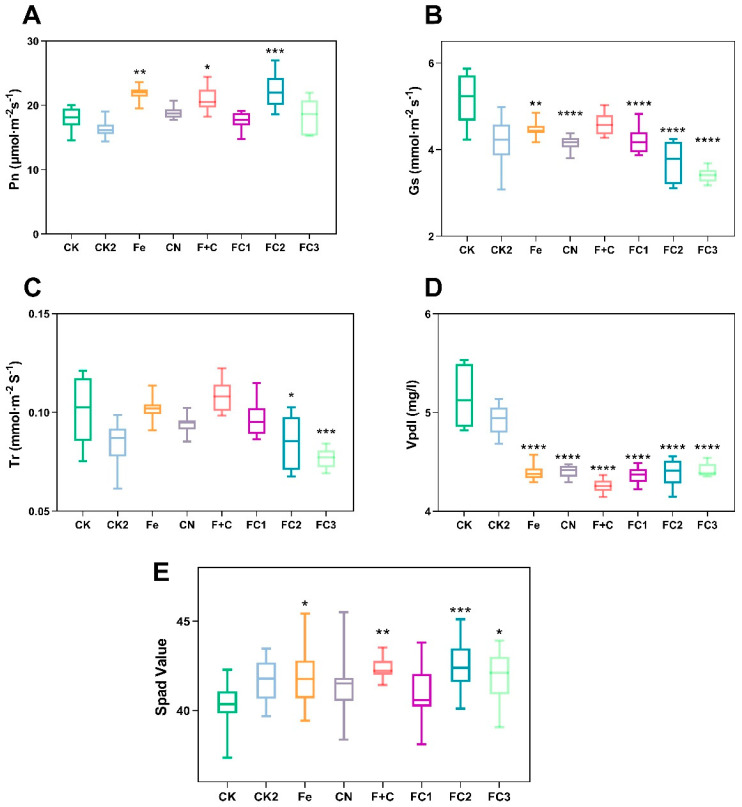
Effects on net photosynthetic rate (**A**), stomatal conductance (**B**), transpiration rate (**C**), and water vapor pressure difference (**D**) in leaves. Relative chlorophyll content (SPAD) (**E**). Asterisks (*) indicate statistically significant differences compared to the control group: * *p* < 0.05, ** *p* < 0.01, *** *p* < 0.001, **** *p* < 0.0001.

Compared to the control and other treatments (Figure 4), the photosynthetic rate (Pn) under FC2 was noticeably greater in Figure 4A, demonstrating enhanced photosynthetic efficiency. Figure 4B reveals that stomatal conductance (Gs) substantially decreased in FC2 and FC3 treatments relative to the control, with FC3 showing the lowest Gs. Similarly, Figure 4C indicates transpiration rate (Tr) was significantly reduced under FC2 and FC3. Figure 4D shows leaf water vapor pressure difference (Vpdl) markedly under FC treatments, particularly FC2 and FC3, likely due to increased transpiration efficiency and optimized stomatal function that improved water retention and nutrient uptake. Figure 4E further demonstrates that FC2 treatment significantly elevated SPAD values (chlorophyll content) compared to other treatments. Notably, despite reduced Gs and Tr in FC groups, Pn increased paradoxically. This apparent discrepancy may be attributed to several compensatory mechanisms that enhance photosynthetic efficiency under restricted gas exchange conditions. Improvements in mesophyll conductance could facilitate greater CO_2_ diffusion from the intercellular air spaces to the chloroplasts, thereby compensating for reduced stomatal aperture. Enhanced gₘ may result from modifications in cell wall properties, such as reduced thickness or increased porosity, which improve CO_2_ permeability across the mesophyll layer [54]. Increased biochemical efficiency of carbon fixation likely plays a critical role. This may involve elevated Rubisco carboxylation capacity, improved activation state, or higher catalytic efficiency of Rubisco, which would allow sustained carbon assimilation even at lower internal CO_2_ concentrations [55]. Additionally, upregulation of chloroplast electron transport chain components (e.g., cytochrome b6f complex) and enhanced ATP synthesis could support higher photosynthetic rates by ensuring sufficient energy supply for RuBP regeneration and carbon metabolism. This suggests FC treatments activated compensatory mechanisms such as improved internal CO_2_ utilization, enhanced carbon fixation pathways, and optimized chloroplast activity. Consequently, plants achieved higher water–use efficiency by minimizing non–essential transpiration while maintaining photosynthetic performance, indicating adaptation potential for water–limited environments. Further research should elucidate the biochemical and molecular pathways enabling this gas exchange–restricted photosynthesis enhancement.

### 3.4. Impact of FC on Antioxidant System

By neutralizing reactive oxygen species (ROS) produced during metabolic processes and environmental challenges, plant antioxidant systems function as vital defense networks against oxidative stress [56,57,58,59]. These systems consist of non–enzymatic antioxidants (such ascorbate, glutathione, and flavonoids) that scavenge free radicals and enzymatic components (like SOD, CAT, and POD) that catalytically detoxify ROS, collectively maintaining cellular redox homeostasis and protecting macromolecules from oxidative damage [60,61,62].

**Figure 5:**
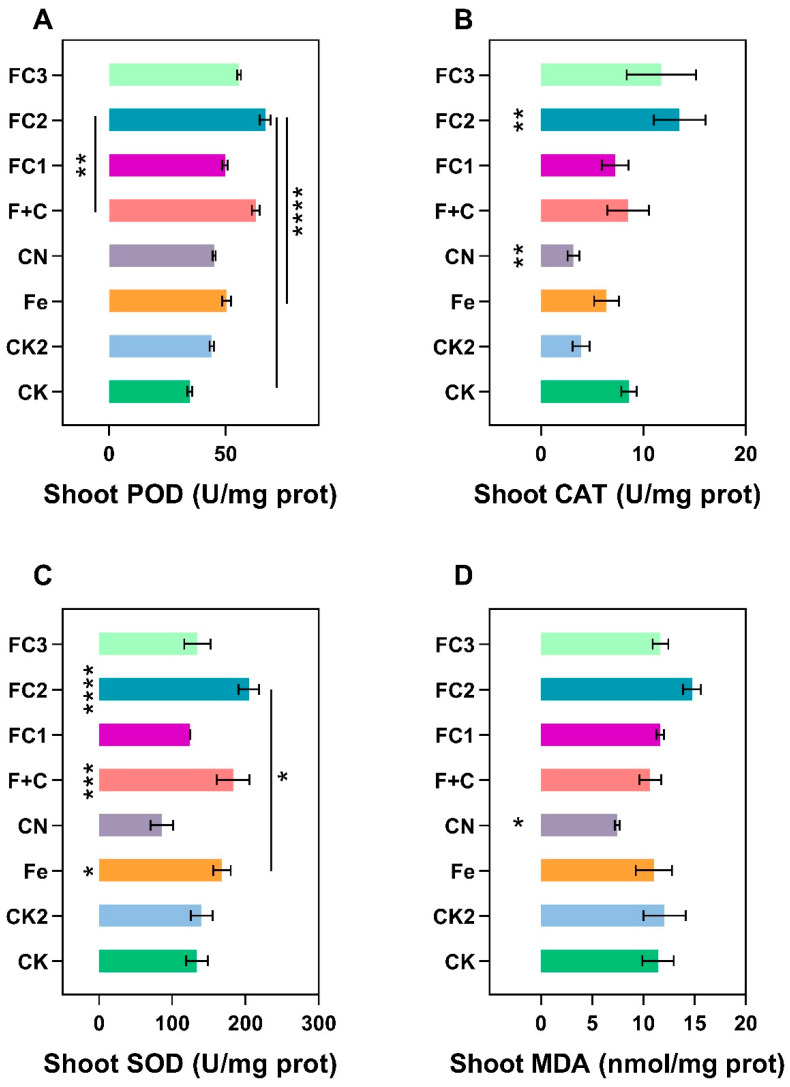

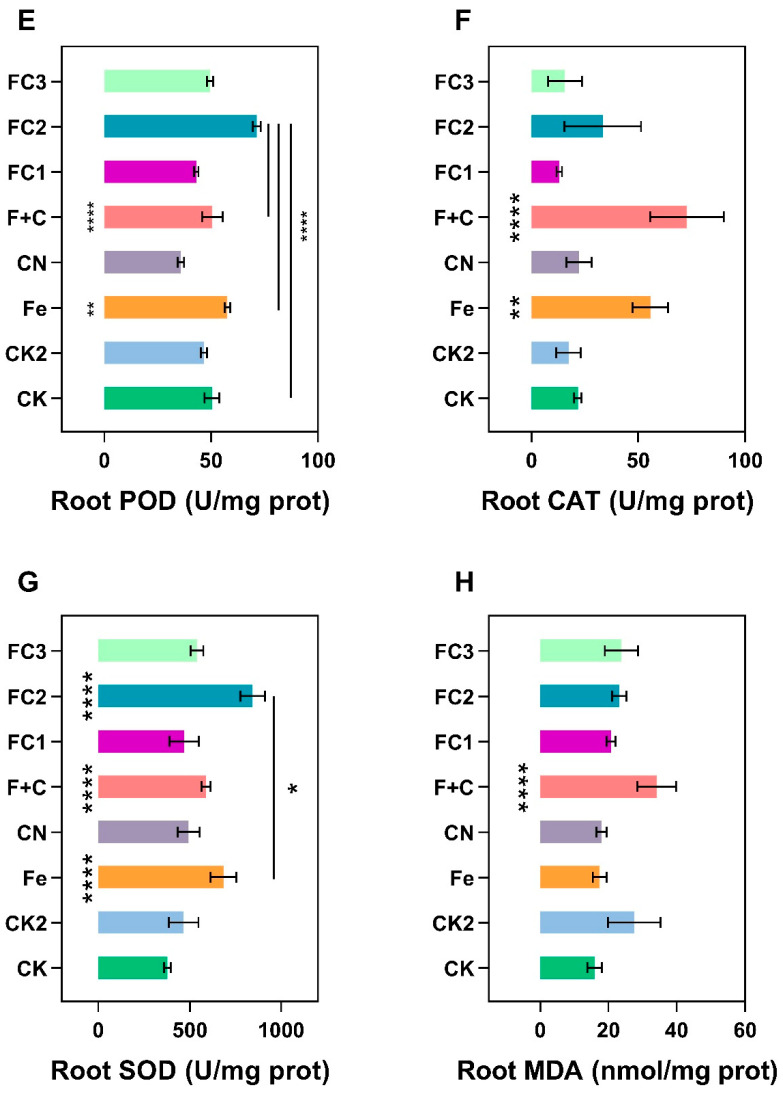
Impact on soybean antioxidant enzyme system. (**A**), Shoot POD activity. (**B**), Shoot CAT activity. (**C**), Shoot SOD activity. (**D**), Shoot MDA content. (**E**), Root PODactivity. (**F**), Root CAT activity. (**G**), Root SOD activity. (**H**), Root MDA content. Asterisks (*) indicate statistically significant differences compared to the control group: * *p* < 0.05, ** *p* < 0.01, *** *p* < 0.001, **** *p* < 0.0001.

Enzymatic activities and oxidative stress markers across treatments are presented in Figure 5. Shoot peroxidase (POD) activity increased significantly under FC treatments relative to control (CK), with FC2 and FC3 showing maximal enhancement (Figure 5A). Root POD similarly increased under FC treatments (Figure 5E). Catalase (CAT) activity in shoots exhibited moderate elevation under FC2 and CN (Figure 5B), while roots displayed maximal CAT activity under F + C, exceeding CK, FC1, and Fe treatments (Figure 5F).

Superoxide dismutase (SOD) activity surged in both shoots and roots under FC2 and FC3, though F + C also induced significant but comparatively smaller increases (Figure 5C,G). Malondialdehyde (MDA) content revealed tissue–specific responses: shoots showed MDA reduction only in CN, whereas roots accumulated highest MDA under F + C (Figure 5D,H). FC treatments consistently maintained lower MDA levels, indicating reduced oxidative damage.

The marked upregulation of key antioxidant enzymes under FC treatments, particularly FC2 and FC3, suggests a potential mitigation of ROS–mediated inhibition in biological nitrogen fixation [63]. ROS can directly inactivate nitrogenase—the oxygen–labile enzyme responsible for N_2_ reduction—by disrupting its iron–sulfur (Fe–S) clusters and oxidizing the iron–molybdenum cofactor (FeMoco), thereby impairing electron transfer and catalytic function [64,65,66]. The observed enzymatic enhancements may thus contribute to a stabilized redox microenvironment within nodules, potentially protecting nitrogenase integrity and prolonging its activity [67]. We further hypothesize that the significant reduction in MDA content—a marker of membrane lipid peroxidation—may reflect an amelioration of oxidative damage, which could foster more favorable conditions for iron homeostasis [68]. Iron is essential not only as a cofactor in nitrogenase but also for electron transport chains and heme biosynthesis in nodules [69,70]. It is therefore plausible that the FC–induced reinforcement of the antioxidant system supports iron metabolism by reducing misallocation or oxidation of iron pools, improving its acquisition, trafficking, and incorporation into metalloproteins [71]. Moreover, the potential synergy between enhanced ROS scavenging and iron utilization remains to be fully elucidated. We propose that FC treatments might facilitate the maintenance of functional iron–sulfur clusters and promote the assembly of nitrogenase subunits under oxidative stress. However, these mechanistic links should be interpreted as hypotheses that require validation through targeted studies measuring real–time nitrogenase activity, Fe–S cluster stability, and iron isotopic tracing under FC exposure. In conclusion, while FC–induced augmentation of antioxidant defenses correlates with improved nitrogen fixation metrics, the explicit mechanisms linking ROS scavenging to nitrogenase protection and iron cofactor utilization remain speculative. These proposed pathways represent testable models for future research into the role of nanomaterial–enhanced plant performance under stress.

### 3.5. Effects on Nutrient Elements

**Figure 6:**
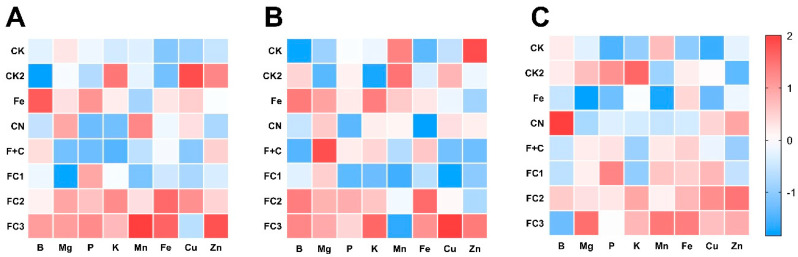
The distribution of standardized content of major mineral elements under different treatments. Roots (**A**), stems (**B**), and leaves (**C**).

In order to profile elemental compositions and assess the effects of different treatments on mineral nutrient absorption and distribution, inductively coupled plasma mass spectrometry (ICP–MS) was utilized [72]. To minimize batch effects, raw ICP–MS data were standardized via z–score transformation, facilitating comparison of relative elemental abundance patterns among treatment groups. The heatmap (Figure 6) provides an overview of elemental content in the roots (Figure 6A), stems (Figure 6B), and leaves (Figure 6C) of plants subjected to various treatments. A visual comparison of nutrient absorption across various treatments and plant sections is made possible by the color scale, where blue denotes lower concentrations and red greater concentrations.

FC treatments (FC1, FC2, FC3) significantly increase iron (Fe) concentration, particularly in roots (Figure 6A) and stems (Figure 6B), with FC3 demonstrating the highest iron accumulation. These findings indicate enhanced iron uptake under FC treatments, especially evident when compared to the control (CK) and Fe–alone treatments where iron levels remain lower. The elevated iron concentrations in roots and stems suggest improved bioavailability or transport mechanisms facilitated by FC treatments.

Beyond iron, magnesium (Mg) levels moderately increase in roots and stems under FC treatments (Figure 6A,B), particularly for FC2 and FC3, implying positive effects on Mg uptake relevant to photosynthesis and cellular functions. Phosphorus (P) concentrations vary across treatments, though FC2 and FC3 show slightly higher P content in roots and stems (Figure 6A,B), indicating enhanced nutrient absorption capacity. Potassium (K) exhibits modest increases in leaves (Figure 6C) under FC treatments, suggesting subtle improvements in leaf nutrient balance.

For micronutrients, both copper (Cu) and zinc (Zn) display elevated concentrations in roots and stems (Figure 6A,B) under FC2 and FC3, reflecting broad nutrient absorption enhancement. Collectively, FC treatments—especially FC3—demonstrate pronounced efficacy in boosting iron absorption in roots and stems while facilitating uptake of magnesium, phosphorus, potassium, copper, and zinc. This contributes to balanced nutrient distribution across plant tissues, indicating that FC treatments not only optimize iron availability but also comprehensively improve nutrient uptake to support plant growth and development.

### 3.6. Effects on Carbon and Nitrogen Content

**Figure 7:**
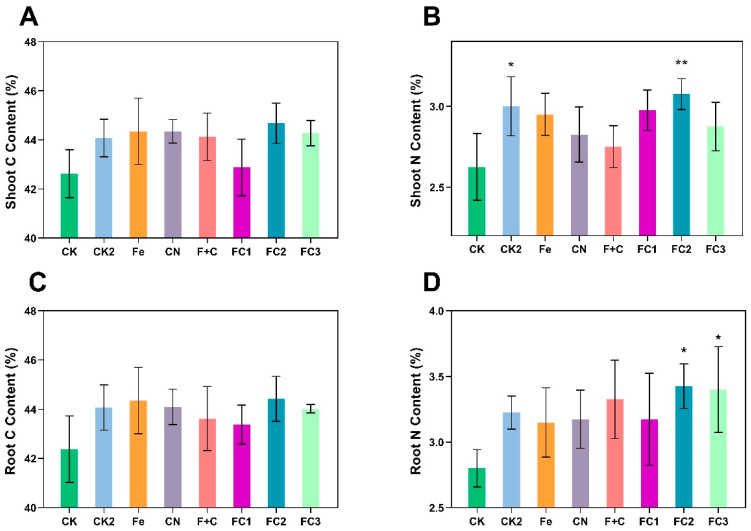
Effects on carbon and nitrogen content in shoot and root. Shoot C Content (**A**). Shoot N Content (**B**). Root C Content (**C**). Root N Content (**D**). Asterisks (*) indicate statistically significant differences compared to the control group: * *p* < 0.05, ** *p* < 0.01, *** *p* < 0.001, **** *p* < 0.0001.

Analysis of carbon (C) and nitrogen (N) content in roots and shoots revealed distinct nutrient dynamics (Figure 7). Shoot C content (Figure 7A) and root C content (Figure 7C) remained statistically unchanged across treatments, despite slight numerical increases in FC–treated plants relative to control (CK). In contrast, shoot N content (Figure 7B) increased significantly in CK2 and FC2, while root N content (Figure 7D) was markedly higher in FC2 and FC3 than in CK. These results indicate that FC treatments minimally influence carbon accumulation but significantly enhance nitrogen content—particularly in roots—suggesting enhanced root–specific nitrogen assimilation or preferential allocation under these conditions.

## 4. Conclusions

This study effectively illustrated FC’s substantial potential as a dual–purpose nanofertilizer to promote soybean growth. The administration of FC produced significant benefits in plant growth, symbiotic nitrogen fixation, and general nutritional status, especially at a dose of 100 mg kg^–1^ (FC2).

Important results show that FC treatments significantly increased the quantity and fresh weight of root nodules, which are essential for nitrogen fixation, as well as shoot and root biomass. The composite’s ability to supply iron was evident from elevated Fe concentrations in plant tissues, which in turn correlated with higher chlorophyll content and net photosynthetic rates. Notably, this photosynthetic enhancement occurred despite reduced stomatal conductance, pointing to a sophisticated improvement in water–use efficiency and internal carbon utilization.

Moreover, the FC composite fortified the plant’s defense mechanisms, as shown by the heightened activity of antioxidant enzymes like POD and SOD, which helps mitigate cellular stress. The fundamental function of the composite was highlighted by the elemental analysis, which showed improved absorption of both iron and nitrogen, both of which are necessary for strong plant development.

In conclusion, the Fe_2_O_3_/g–C_3_N_4_ nanocomposite demonstrates a role beyond that of a simple nutrient carrier by actively enhancing the physiological and metabolic processes of soybeans during the critical vegetative growth phase. Our findings indicate that by providing a slow–release source of iron and stimulating the plant’s innate nitrogen–fixing capabilities, this nanocomposite holds potential as a tool for sustainable agriculture. The observed improvements in early biomass accumulation and nodulation are valuable indicators of enhanced plant vigor and physiological efficiency. However, as this study captured effects within a 60–day experimental period focusing on early vegetative growth and nodulation, it does not fully assess reproductive outcomes or direct yield parameters. To confirm these results and maximize utility for broad agricultural usage, more field–scale research is necessary.

## Figures and Tables

**Table 1:** The soil physicochemical properties [22].

Indicator	Soil (Average Value)
pH	8.38
Total organic matter (g kg^−1^)	8.36
Available potassium (AK) (mg kg^−1^)	66.07
Available phosphorus (AP) (mg kg^−1^)	11.76
Available Nitrogen (AN) (mg kg^−1^)	20.37
Available Fe (mg kg^−1^)	22.91
Electrical conductivity (dS m^−1^)	0.16
Organic matter (g kg^−1^)	11.31

## Data Availability

The datasets presented in this article are not easily made public due to confidentiality agreements. Requests to access the datasets should be directed to the first author.
